# Detection of iodixanol-induced allergic reaction signals in Chinese inpatients: a multi-center retrospective database study using prescription sequence symmetry analysis

**DOI:** 10.3389/fphar.2024.1298021

**Published:** 2024-03-27

**Authors:** Dandan Zhang, Xinchen Yang, Zhangwei Yang, Wei Sun, Shunjie Chen, Lingxiao Xu

**Affiliations:** ^1^ Department of Pharmacy, Shanghai Pulmonary Hospital, School of Medicine, Tongji University, Shanghai, China; ^2^ Electric Engineering and Automation, East China University of Science and Technology, Shanghai, China; ^3^ Medical Department, Shanghai Fourth People’s Hospital, School of Medicine, Tongji University, Shanghai, China; ^4^ Health Statistics Teaching and Research Section, Tongji University, Shanghai, China

**Keywords:** iodixanol, allergic reactions, multi-center study, prescription sequence symmetry analysis, Chinese inpatients

## Abstract

**Objective:**

This study aimed to explore the signal detection method for allergic reactions induced by inpatient iodixanol injection.

**Methods:**

A database of 3,719,217 hospitalized patients from 20 large Chinese general hospitals was processed and analyzed using the prescription sequence symmetry analysis (PSSA) method.

**Results:**

126,680 inpatients who used iodixanol and were concurrently treated with anti-allergic drugs were analyzed. In the medical records of these patients, only 32 had documented iodixanol allergies. Statistical analysis identified 22 drugs in 4 categories—calcium preparations, adrenergic/dopaminergic agents, glucocorticoids, and antihistamines—as marker drugs. With time intervals of 3, 7, and 28 days, the adjusted sequence ratios (aSRs) for all anti-allergics and the 4 categories were greater than 1. The 7-day aSRs were 2.12 (95% CI: 2.08–2.15), 1.70 (95% CI: 1.68–1.73), 3.85 (95% confidence interval [CI]: 3.75–2.30), 2.30 (95% CI: 2.26–2.35), and 1.95 (95% CI: 1.89–2.02), respectively. The proportions of adverse drug events indicated by each signal were as follows: all anti-allergics (2.92%–3%), calcium gluconate (0.19%–0.52%), adrenergic/dopaminergic agents (2.20%–3.37%), glucocorticoids (3.13%–3.76%), and antihistamines (1.05%–1.32%).

**Conclusion:**

This first multi-center Chinese inpatient database study detected iodixanol-induced allergy signals, revealing that reactions may be much higher than those in collected spontaneous reports. Iodixanol risk exposure was closer to actual pharmaceutical care findings. PSSA application with ≤7-day intervals appears better suited for monitoring late allergic reaction signals with these drugs.

## 1 Introduction

Iodixanol is a widely used intravenous non-ionic dimeric iodine contrast agent. Its isotonicity with plasma, high safety profile, and minimal impairment of renal function make it well suited as an adjunct for clinical diagnostic imaging, interventions, vascular stenting, and surgical treatments. In the first half of 2021, China ranked number one globally in the consumption of iodixanol injection (China Industrial Research Network, 2022). Concurrently, reported adverse drug reaction (ADR) cases caused by iodixanol have significantly increased. A total of 20,185 patients who received contrast iodixanol were recruited from 95 medical centers in China (Zhang et al., 2014). The immediate adverse reactions within 1 h of administration and the delayed adverse reactions from 1 h to 7 days after administration were recorded. The overall iodixanol-induced adverse reaction rate was 1.52%, with immediate reactions accounting for 0.58% and delayed reactions accounting for 0.97%. The major delayed reactions were mild and mostly occurred on the skin (0.68%), including rash, pruritus, and urticaria. A Korean meta-analysis found an allergic reaction rate of 0.85% (Suh et al., 2019). The majority of reports are sourced through the National ADR Monitoring Network or voluntary submissions from hospitals. When clinical adverse drug events (ADEs) occur, specifics such as allergic reactions may be documented in the electronic medical record (EMR) fields of hospital records, facilitating retrospective data analysis. Nevertheless, underreporting poses a significant challenge within voluntary systems. Systematic reviews reveal a median underreporting rate of 94% for spontaneous reporting on a global scale (Hazell and Shakir, 2006). Real-world ADEs are likely to surpass the officially reported outcomes. Meanwhile, an ongoing pharmaceutical care study, involving the simultaneous observation of 415 patients and comprehensive documentation, revealed a 30.64% overall incidence of iodixanol-induced ADEs (Zhang et al., 2022). This study found that immediate reactions accounted for 14.55%, delayed reactions accounted for 85.45%, mild reactions accounted for 73.64%, moderate reactions accounted for 25.45%, and severe reactions accounted for 0.91%. Thus, re-evaluating safety and efficacy relying solely on passively collected ADRs may not be efficient at uncovering potential risks due to extensive unreported data.

With the advent of big data, real-world study (RWS)-based drug safety re-evaluation utilizing active monitoring has become more common. RWS data sources derive mainly from collecting, processing, statistically analyzing, and scientifically interpreting EMRs. This elevates RWS to real-world evidence (RWE). Prescription sequence symmetry analysis (PSSA) is one RWS drug safety signal mining technique using large medical databases. It rapidly identifies adverse event signals and potential prescribing cascades. PSSA assumes that adverse reactions to drugs prompt prescriptions for other drugs (marker drugs). Therefore, patient records exhibit specific temporal exposure and marker drug frequency distributions (Tao and Zhan, 2012). A systematic literature review found that PSSA is widely used internationally and considered highly suitable for active adverse reaction surveillance. Recently, China has also begun utilizing PSSA on large databases while conducting methodological summaries (He et al., 2021; Lyu et al., 2021; Morris et al., 2022). Compared to traditional epidemiology, PSSA better controls time-invariant confounding factors and requires few variables to complete signal mining. This enables rapid, accurate, and low-cost detection (Zhou et al., 2019). To expand the PSSA methodology, this study mined multi-center data to uncover iodixanol allergic reaction signal characteristics and influencing factors.

## 2 Data and methods

### 2.1 Data source

Data were obtained from a large multi-center general hospital database from several Chinese provinces and cities since 2015, detailed previously (Nie et al., 2018). The top 20 hospitals by iodixanol volume were selected, with patient discharges from 1 January 2015 to 31 December 2017. The dataset contained basic inpatient demographics, clinical diagnoses per International Classification of Diseases 10th edition (ICD-10) codes, charges, standardized drug names, and Anatomical Therapeutic Chemical (ATC) classifications. As a retrospective, anonymous, non-interventional analysis, all data were only used for research. The Ethics Committee of Shanghai Fourth People’s Hospital, Tongji University School of Medicine, waived review and consent.

### 2.2 Index and marker drugs

PSSA was applied to monitor iodixanol-induced ADEs by investigating associations between this drug and related therapeutic drugs. PSSA assumes the propensity to initiate a marker drug (e.g., thyroxine) and follows an index drug (e.g., amiodarone) (Chen et al., 2022). There is a greater tendency to start labeled drugs (marker drugs like thyroxine) after versus before index drugs (index drugs like amiodarone). The index drug purportedly causes a side effect (hypothyroidism) when treated by the marker drug. Theoretically, if the index–marker causal relationship is absent, the marker drug use would symmetrically (randomly) occur before and after the index drug. Conversely, if the index drug necessitates marker drug treatment for an ADE, the marker drug would asymmetrically initiate after more often than before the index drug. Recording inpatient drug orders chronologically allows determining the index–marker sequence by timing.

According to the Chinese Expert Consensus on Adverse Reactions Associated with Iodine Contrast Angiography Applications (Chen et al., 2014), iodine contrast adverse reaction timing is classified as acute (within 1 h), delayed (1–7 days), or late (1+ week). Like its analogs, iodixanol predominantly causes allergic reactions, with an overall rate of 0.74%–1.52%. Delayed reactions predominate over acute reactions, with most skin reactions occurring 1 h–2 days after injection and resolving within 1–7 days. However, some reactions have occurred up to 4 weeks later (Häussler, 2010; Tasker et al., 2019). Aside from treatments like oxygen and hydration, iodixanol allergy can also be treated with drugs, including epinephrine, adrenaline class of pressors, glucocorticoids, antihistamines, and calcium (Chen et al., 2014; Zhang et al., 2014). Although inpatient PSSA is less common than long-term outpatient monitoring, observed inpatient data exhibit similar temporal characteristics. Thus, this study designated iodixanol as the index drug and the above therapeutic medications as marker drugs, exploring different hospitalization lengths as the observation period. Since allergic reaction treatments usually involve multiple and rotating drug classes, this study categorized anti-allergics by the first four or five ATC codes. Possible drugs like loratadine and diphenhydramine were aggregated by generic names, regardless of the manufacturer or dosage, as marker drugs to assess signal detection across therapeutic drug classes for potential iodixanol allergic reactions.

### 2.3 Interval and washout period

The PSSA methodology requires determining the index drug treatment interval and corresponding signal detection interval. This entails defining washout and interval periods. The washout period excludes previous users to select new users of index drugs. The interval is the maximum absolute time difference between index and marker drug initiation. The included patients were all inpatients, so each admission was considered a new drug user. Patients prescribed iodixanol in outpatient/emergency settings were excluded. Hence, samples with iodixanol on day 1 or 2 of hospitalization or total stays ≤3 days were excluded. Based on iodixanol-induced allergic reaction clinical occurrence and treatment patterns (Häussler, 2010; Chen et al., 2014), the washout period was 30 days before and after iodixanol use. Signal characteristics were observed at 3-, 7-, and 28-day intervals.

### 2.4 Calculation method of the sequence ratio

Following the PSSA summary by Morris et al. (2022), the analysis entailed four steps:

(1) The crude sequence ratio (cSR) assumed iodixanol as the index drug (I) and anti-allergy drug as the marker drug (M). I and M records were prescribed and used at different times or concurrently. Patients were grouped into “causal” and “non-causal” cohorts based on I and M chronological order. The causal cohort received the index drug I before the marker drug M, and n_index→marker_ was defined. The non-causal cohort received M before I, and n_marker→index_ was defined. The cSR was the total causal cohort samples divided by the non-causal samples:

$$\text{cSR}=\frac{n_{\text{index}\to \text{marker}}}{n_{\text{marker}\to \text{index}}}.$$

(2) The null-effect sequence ratio (neSR) was calculated. Real-world prescriptions can be impacted by various factors like insurance policies, illnesses, and other medications. To adjust for this bias, PSSA calculates the overall weighted probability P:

$$P=\frac{\sum_{m=1}^{\mu }\left[I_{m}\times \left(\sum_{n=m+1}^{m+d}M_{n}\right)\right]}{\sum_{m=1}^{\mu }\left[I_{m}\times \left(\sum_{n=m-d}^{m-1}M_{n}+\sum_{n=m+1}^{m+d}M_{n}\right)\right]},$$

where m is the specific iodixanol (index drug) use date; μ is the predefined hospitalization length post-iodixanol (last survey day), set as 30 days; I_m_ is the number of patients receiving iodixanol first on date m; d is the index–marker time interval; n is the consecutive study days; and M_n_ is the number of patients starting the marker drug on a given day.

After obtaining P, the approximate upper- and lower-interval probability formula for the 95% confidence interval (95% CI) of the overall binomial distribution rate when n > 200 and P × n > 15 is (Liu, 2004)

$$P_{\alpha }\approx \left(P\pm Z_{\alpha /2}\sqrt[{2}]{P\left(1-P\right)/n}\right),$$

where n is the final sample size. Z_α/2_ is 1.96 for a 95% two-sided alpha test.

The neSR is then obtained by the given equation:

$$\text{neSR}=\frac{P_{\alpha }}{1-P_{\alpha }}.$$

(3) The adjusted sequence ratio (aSR) was

$$\text{aSR}=\frac{\text{cSR}}{\text{neSR}}.$$

The aSR was obtained by the cSR/neSR, an adjusted sequence ratio obtained after excluding possible confounding factors. When the lower 95% CI of the aSR was greater than 1, it indicated a possible causal association between the index drug and ADR.

(4) The excess risk among exposed adjusted (ERAEA) for significant signal drugs (lower-confidence interval aSR > 1) was estimated as

$$\text{ERAEA}=\frac{n_{\text{index}\to \text{marker}}∙\frac{\left(\text{aSR}-1\right)}{\text{aSR}}}{n_{\text{index}}},$$

where n_index→marker_ refers to patients who used the index drug after marker drugs and n_index_ is the total index drug users.

### 2.5 Data processing and statistical methods

In this study, PL/SQL was used as the pre-data processing and terminal access tool, based on ORACLE 11g for pre-data processing. The post-data were developed using .net (version 2013) software, by which special software was written for data processing. Furthermore, intermediate and feature tables were constructed, and IBM SPSS 22.0 statistical software was used for statistical analysis of the study data. The count data were expressed as rates (%) expressed by the Χ^2^ test. A statistically significant difference was considered at *p* < 0. 05.

The minimum sample size estimate was

$$n=\frac{{\left(z_{\alpha /2}\right)}^{2}\times \pi \left(1-\pi \right)}{E^{2}},$$

where Z_α/2_ is the two-sided alpha test table value, π is the assumed incidence rate, and E is the tolerance error, generally half the confidence interval width. A 0.85% allergic reaction proportion meta-analysis was assumed (Suh et al., 2019). The confidence interval was 95%, making Z_α/2_ 1.96. Another study provided incidence bounds of 0.36%–1.95%, giving E = (0.0036 + 0.0195)/2. The minimum sample size was

$$n=\left(1.962\right)\times 0.0085\times \left(1-0.0085\right)/\left(0.0036+0.0195\right)/\left.2\right)2\approx 243.$$

## 3 Results

### 3.1 Patient inclusion and exclusion

**FIGURE 1:**
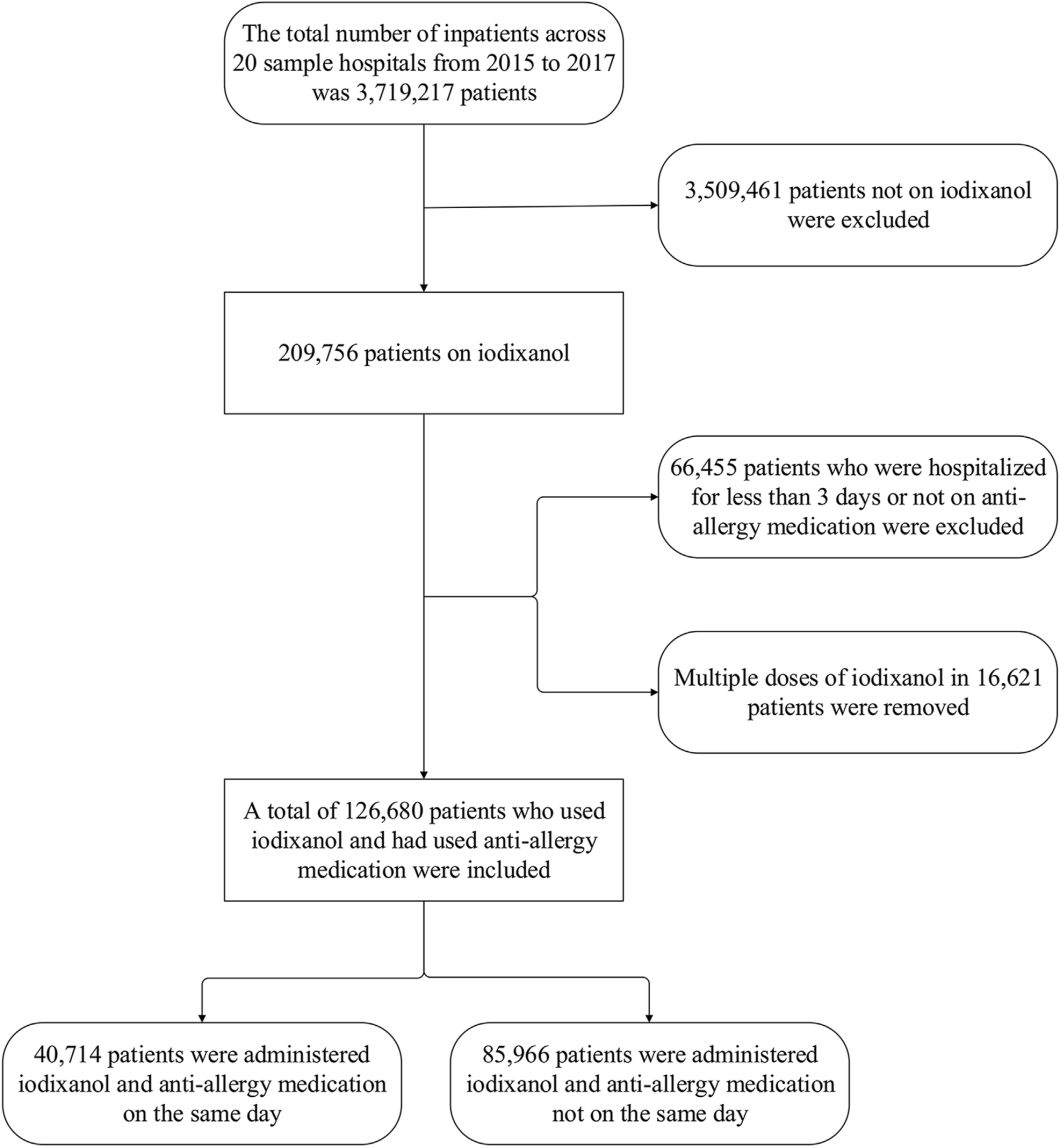
Population screening flow chart for iodixanol application with prescription sequence symmetry analysis..

Figure 1 displays the sample screening. The 20 hospitals had 3,719,217 inpatients during 2015–2017, with 209,756 (5.64%) using iodixanol. Of the total number of patients, 126,680 were eventually included. Then, 83,076 used iodixanol but were hospitalized <3 days or had not used anti-allergics.

Table 1 outlines the characteristics of the included patients. Among the 126,680 patients considered, 64.1% were males, representing a higher proportion than females. Examining the age distribution, adults aged 18–65 years accounted for 62.16%, the elderly over 65 years old constituted 36.79%, and minors under 18 years comprised only 1.05%. The top five primary diagnoses among inpatients collectively made up 55.96% of the entire enrolled population. These diagnoses, in descending order, were ischemic heart disease, cerebrovascular disease, gallbladder, biliary tract and pancreatic disorders, malignant neoplasm of the digestive organs, and special operations and healthcare. Notably, surgical patients accounted for 80.75% of this subset. In the medical record home pages, only 8.08% of patients had a documented history of allergies or allergic reactions. Specifically, regarding allergy records, 0.16% showed documented iodine contrast allergy, although the specific preparation was not clear. Only 32 cases of documented iodixanol allergy were recorded, constituting 0.03% of the total patient population.

**TABLE 1:** Characteristics of inpatients receiving iodixanol included in this study (*n* = 126,680).

Category	Characteristics	Study population	Constituent ratio (%)	*p*-value
Gender	Male	81,199	64.1	<0.001
	Female	45,481	35.9	
Age (years)	<18	1,327	1.05	<0.001
	≥18 and <65	78,744	62.16	
	≥65	46,609	36.79	
Top 5 rankings of major diagnoses	Ischemic heart disease (I20–I25)	36,373	28.71	<0.001
	Cerebrovascular disease (I60–I69)	17,152	13.54	
	Gallbladder, biliary tract, and pancreatic disorders (K80–K87)	6,386	5.04	
	Malignant neoplasm of the digestive organs (C15–C26)	5,722	4.52	
	Special operations and healthcare (Z40–Z54)	5,253	4.15	
To operate or not	Surgery	102,295	80.75	<0.001
	Non-surgical	14,764	11.65	
	Missing data	9,621	7.59	
Allergy records	No history of allergies	116,441	91.92	<0.001
	History of allergies	10,239	8.08	
	History of allergies to iodine preparations or contrast media	199	0.16	0.381
	Iodixanol	32	0.03	

Notes: *Pearson’s chi-square test (Χ^2^) using two-sided test results.

### 3.2 Marker drug use

Further analysis was performed on marker drugs treating allergic reaction symptoms. Iodixanol users had 31 anti-allergic drug varieties. Ketotifen, levocetirizine, cyproheptadine, tretinoin, imipramine, midodrine, fexofenadine, beclomethasone, and Avastin ranked in the bottom 9 by usage, with <235 users each. Table 2 shows the ranking, number of hospitals, and usage proportions for the other 22 varieties. Among the anti-allergic drugs, the availability of different preparations varied across the 20 hospitals. Some formulations, such as dimenhydrinate and methoxamine, were less commonly used. Notably, the most frequently utilized preparations included dexamethasone, methylprednisolone, dopamine, promethazine, and noradrenaline. The data showed that over 50% of patients received prescriptions for methylprednisolone or noradrenaline concurrently with iodixanol on the same day. Following these, the next most commonly administered drugs were dexamethasone and metaramine, both of which are available in injectable formulations.

**TABLE 2:** Ranking of inpatients who received anti-allergic medication and iodixanol (*n* = 126,680).

No.	Anti-allergic drug	Hospitals	Users (n/%)	Same-day usage (n/%)	Injections (%)
1	Dexamethasone	20	39,205 (30.95)	12,334 (31.46)	97.36
2	Methylprednisolone	20	14,293 (11.28)	8,208 (57.43)	100
3	Dopamine	20	13,466 (10.63)	3,561 (26.44)	100
4	Promethazine	20	9,361 (7.39)	1,507 (16.10)	98.99
5	Noradrenaline	20	9,108 (7.19)	4,759 (52.25)	100
6	Calcium gluconate	20	7,969 (6.29)	638 (8.01)	100
7	Phenylephrine	16	6,803 (5.37)	1,168 (17.17)	100
8	Adrenaline	19	5,331 (4.21)	886 (16.62)	100
9	Prednisolone	14	4,047 (3.19)	975 (24.09)	100
10	Loratadine	16	2,221 (1.75)	212 (9.55)	0
11	Metaradrine	20	2,026 (1.6)	729 (35.98)	100
12	Prednisone	20	1,630 (1.29)	123 (7.55)	0
13	Isoprenaline	20	1,576 (1.24)	247 (15.67)	100
14	Hydrocortisone	20	1,110 (0.88)	128 (11.53)	100
15	Dobutamine	19	1,073 (0.85)	50 (4.66)	100
16	Desloratadine	9	907 (0.72)	86 (9.48)	0
17	Diphenhydramine	8	701 (0.55)	131 (18.69)	98.75
18	Chlorpheniramine	16	555 (0.44)	62 (11.17)	0
19	Cetirizine	11	473 (0.37)	47 (9.94)	0
20	Ebastine	12	446 (0.35)	31 (6.95)	0
21	Dimenhydrinate	4	439 (0.35)	34 (7.74)	0
22	Methoxamedrine	6	278 (0.22)	29 (10.43)	0

**FIGURE 2:**
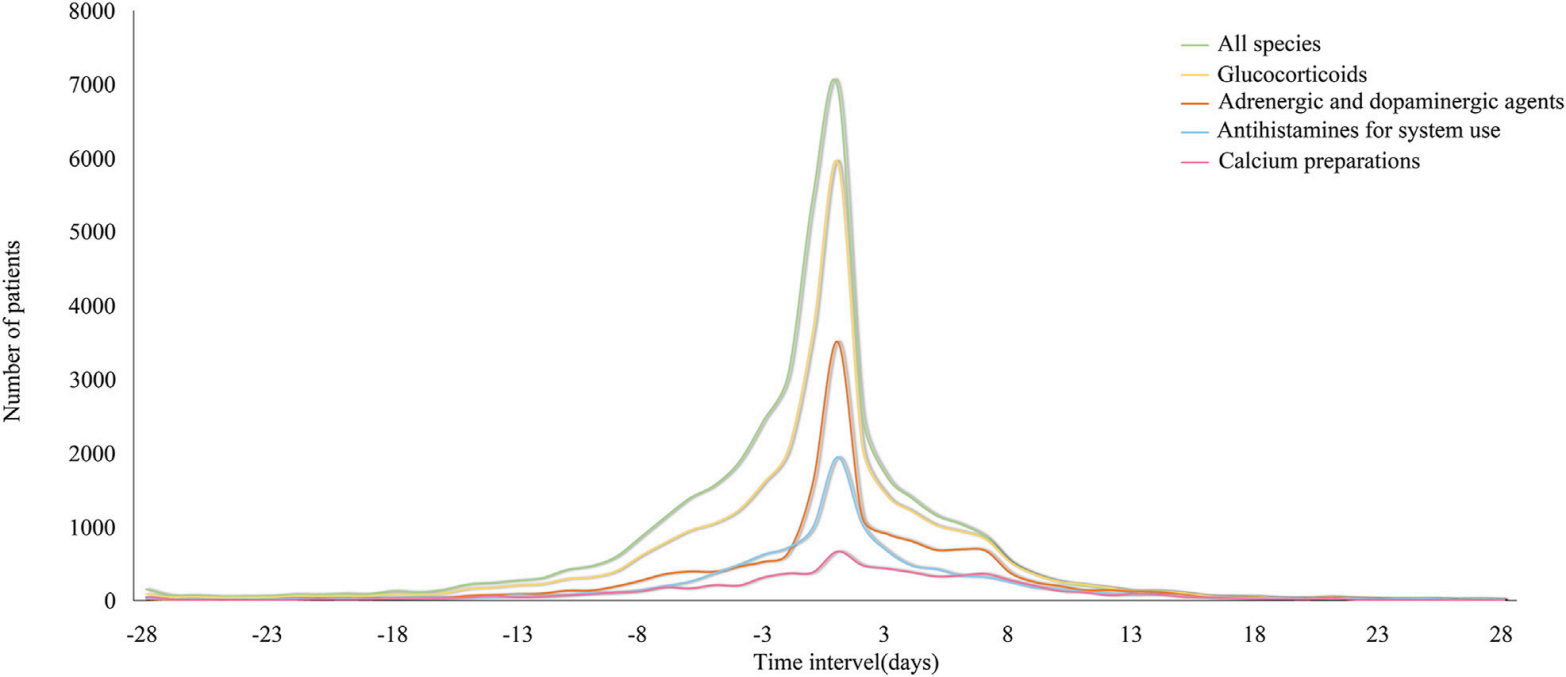
Distribution of inpatients’ anti-allergic drugs count by category before and after iodixanol.

Figure 2 illustrates the distribution of these drugs by ATC classification (A12AA, C01CA, H02AB, R06A, and all combined) before and after the administration of iodixanol, simulating the normal distribution map of the various classes. In general, a right skew was evident both before and after the use of iodixanol, with a gradual downward trend observed on days 3–7. Notably, the use of glucocorticoids, adrenergic, and dopaminergic agents did not experience a sharp decrease until approximately day 7.

### 3.3 Individual marker drug-adjusted sequence ratios

Using the anti-allergics given in Table 2 as marker drugs, adjusted sequence ratios were calculated for treating potential iodixanol-induced allergic reactions. The results are presented in Table 3, indicating that, among potential iodixanol allergic reactions, only prednisone (3-day, aSR < 1) showed a sequence ratio below 1. For all four drug classes—calcium channel blockers, adrenergic/dopaminergic agents, glucocorticoids, and antihistamines—the aSR exceeded 1, irrespective of the category or individual drug.

**TABLE 3:** Results of prescription sequence symmetry analysis on marker drugs for allergic reactions induced by iodixanol.

Category	Marking of drugs/interval	Iodixanol pre-/post-medication (N)			aSR (95% CI)		
		3 d	7 d	28 d	3 d	7 d	28 d
Overall		11,913/16,019	15,916/19,681	17,890/21,528	2.12 (2.08–2.15)	1.70 (1.68–1.73)	1.51 (1.49–1.54)
A12AA, calcium preparations	Calcium gluconate	1,663/1,832	3,047/2,369	4,372/2,839	1.31 (1.25–1.37)	1.39 (1.33–1.45)	1.32 (1.27–1.39)
C01CA, adrenergic and dopaminergic agents		5,701/3,486	8,527/4,831	10,311/5,839	5.22 (5.08–5.38)	3.85 (3.75–3.95)	3.18 (3.10–3.26)
	Dopamine	3,496/2,363	5,248/2,943	6,445/3,334	3.44 (3.31–3.57)	2.90 (2.80–3.00)	2.57 (2.48–2.66)
	Noradrenaline	1,362/588	2,146/930	3,019/1,227	12.1 (11.5–12.8)	7.55 (7.19–7.93)	5.60 (5.36–5.86)
	Phenylephrine	2,363/175	3,737/434	4,819/760	21.6 (20.6–22.7)	9.09 (8.67–9.54)	5.38 (5.13–5.65)
	Adrenaline	1,038/886	1,987/1,150	2,995/1,346	3.34 (3.14–3.56)	2.64 (2.50–2.79)	2.27 (2.15–2.40)
	Metaradrine	209/319	468/461	667/603	3.08 (2.76–3.47)	2.77 (2.51–3.06)	2.31 (2.11–2.54)
	Isoprenaline	337/124	761/178	1,086/229	5.50 (4.95–6.12)	4.07 (3.68–4.49)	3.25 (2.94–3.60)
	Dobutamine	248/104	531/163	788/208	2.70 (2.39–3.05)	2.54 (2.25–2.87)	2.20 (1.94–2.49)
	Methoxamedrine	71/28	116/51	171/69	5.87 (4.58–7.74)	3.66 (2.88–4.73)	2.75 (2.17–3.51)
H02AB, glucocorticoids		9,916/1,0405	13,700/12,993	15,764/14,624	2.95 (2.89–3.01)	2.30 (2.26–2.35)	2.00 (1.96–2.03)
	Dexamethasone	9,177/8,315	12,697/10,477	14,827/11,859	3.11 (3.04–3.19)	2.43 (2.38–2.48)	2.09 (2.05–2.13)
	Methylprednisolone	1,475/1,735	2,396/2,349	3,079/2,886	4.55 (4.35–4.77)	3.48 (3.34–3.62)	2.93 (2.83–3.05)
	Prednisolone	957/271	1,906/418	2,406/630	8.05 (7.53–8.61)	5.54 (5.21–5.90)	3.92 (3.69–4.17)
	Prednisone	311/441	609/496	931/527	0.97 (0.88–1.07)	1.30 (1.18–1.43)	1.35 (1.22–1.49)
	Hydrocortisone	259/161	426/244	616/343	2.99 (2.64–3.39)	2.39 (2.12–2.70)	1.90 (1.69–2.14)
R06A, antihistamines for system use		3,777/3,035	5,281/4,050	6,593/4,626	2.39 (2.30–2.48)	1.95 (1.89–2.02)	1.72 (1.66–1.78)
	Promethazine	2,270/2,098	3,296/2,938	4,305/3,425	2.74 (2.62–2.87)	2.08 (2.00–2.17)	1.78 (1.71–1.86)
	Loratadine	990/322	1,316/388	1,547/429	3.09 (2.84–3.36)	2.82 (2.59–3.06)	2.60 (2.39–2.83)
	Desloratadine	394/214	493/233	555/248	1.97 (1.73–2.25)	1.94 (1.70–2.21)	1.86 (1.62–2.12)
	Diphenhydramine	125/160	207/228	274/272	2.66 (2.24–3.21)	2.09 (1.78–2.48)	1.75 (1.50–2.06)
	Chlorpheniramine	204/109	285/129	333/146	2.41 (2.04–2.87)	2.34 (1.98–2.78)	2.18 (1.84–2.58)
	Cetirizine	186/103	237/122	283/136	2.08 (1.73–2.50)	1.98 (1.65–2.38)	1.85 (1.54–2.22)
	Ebastine	155/99	237/110	289/119	1.61 (1.34–1.95)	1.74 (1.44–2.10)	1.70 (1.40–2.05)
	Dimenhydrinate	109/118	169/150	241/161	1.56 (1.29–1.91)	1.32 (1.09–1.60)	1.28 (1.06–1.55)

Note: aSR, adjusted sequence ratio.

### 3.4 Excess risk among exposed adjusted

Table 4 provides the excess risk among exposed adjusted extrapolated for all drugs and categories.

**TABLE 4:** Excess risk among the exposed adjusted and estimated population of drugs used in the treatment of allergic reactions to iodixanol (*n* = 209,756).

Category and time interval	Adjusted percentage of additional risk exposure	Adjusted number of additional risk exposure	Percentage of the total population (%)
Overall			
3 d	0.10	6,294	3.00
7 d	0.10	6,607	3.15
28 d	0.09	6,108	2.91
A12AA, calcium preparation			
3 d	0.05	396	0.19
7 d	0.11	864	0.41
28 d	0.14	1,085	0.52
C01CA, adrenergic and dopaminergic agents			
3 d	0.18	4,611	2.20
7 d	0.24	6,313	3.01
28 d	0.27	7,075	3.37
H02AB, glucocorticoids			
3 d	0.13	6,557	3.13
7 d	0.15	7,766	3.70
28 d	0.16	7,894	3.76
R06A, antihistamines for system use			
3 d	0.17	2,198	1.05
7 d	0.20	2,585	1.23
28 d	0.21	2,777	1.32

## 4 Discussion

Approximately 75 million CT scans are conducted each year in the United States, and half of them include the use of iodinated contrast media (ICM). In Korea, it is estimated that more than 4 million CT scans involve ICM, but the proportion of iodixanol used in large sample databases and the rate of spontaneous reporting of ADRs are unclear (Cha et al., 2019). Based on the results of this study, we can calculate that the proportion of iodixanol used was 5.64% (209,756/3,719,217). Therefore, it can be inferred that among the 92.98 million patients admitted to 2,548 tertiary Chinese hospitals in 2018 (Ministry of Health of China, 2022), over 5 million inpatients may receive iodixanol annually.

Since the occurrence of allergic reactions and subsequent treatment drugs following iodixanol are unclear, we included potential reaction treatment drug categories and varieties according to the literature and guidelines as index medications. Glucocorticoids have anti-inflammatory, immunosuppressive, anti-shock, and other effects and are widely used clinically, but they lack specificity for allergic reactions. Antihistamines are commonly used allergy drugs exhibiting strong specificity and having a sufficient sample size. Calcium agents are commonly used adjuvant drugs for allergic reactions, showing some specificity. Epinephrine is mainly used for severe allergic reactions and can serve as a marker for such reactions (Chen et al., 2014; Chen et al., 2014; Kuna et al., 2016; Kuna et al., 2016; Huang et al., 2022; Huang et al., 2022). Resulting allergy therapies encompassed systemic glucocorticoids like dexamethasone and methylprednisolone; adrenergic/dopaminergic agents such as dopamine, noradrenaline, and phenylephrine; calcium gluconate; and antihistamines, including promethazine and loratadine. These are commonly hospital-administered. Methylprednisolone, noradrenaline, and iodixanol have high same-day application rates (Table 2). We presume that iodixanol is primarily used for cardiovascular/cerebrovascular diagnosis, often paired with prompt surgery and multidrug treatment. Some patients may also receive glucocorticoids like dexamethasone with iodixanol to prevent reactions, as per early guidelines and the literature (Chen et al., 2014; He, 2017). Figure 1 shows anaphylactic treatments given with the iodixanol concentrate within 7 days. This conforms to most allergic reactions arising within 7 days, especially acute reactions within 1 h. It should be noted that PSSA does not account for index and marker drugs administered concurrently, which could miss signals for acute and severe reactions like shock, which are more often treated with epinephrine. However, including the index–marker sequential order on the same day would confer the same bias.

This demonstrates allergy signal detection capacity. Three-day signal detection exceeded 7 or 28 days, reflecting reaction patterns. Phenylephrine, noradrenaline, and prednisolone have relatively high aSRs, fitting their emergency acute reaction treatment use. However, considering that the typical medical administration of iodixanol within days does not match acute reaction timing, signal interference from illness or other treatments is also plausible. Adding a matched blank control cohort could improve this. Antihistamines also showed the strongest detection with the highest aSR for loratadine. This conforms to their oral preparations and delayed reaction treatment applications.

Although most literature records suggest that immediate and non-immediate hypersensitivity reactions to ICM occur at a frequency of 0.5%–3% in patients receiving non-ionic ICM (Torres et al., 2021), ADRs are highly likely to be underreported. From 2009 to 2017, only 2,469 cases of ADRs were collected from nearly 200 hospitals in the region, of which iodixanol ADRs ranked first (533, 42.30%), with rash, pruritus, and flushing as the top 3 reactions. Furthermore, 90.48% of ADRs occurred within 24 h (Xu et al., 2020). Thus, the proportion of spontaneous reporting records was only 0.035%, consistent with this study. Although active pharmacological care can detect missed adverse events (Hu et al., 2022), it is less efficient and labor-intensive. Table 4 shows that the ADE proportion estimates based on excess risk among exposed adjusted were 3.00%–2.92% for all anti-allergics combined, 0.19%–0.52% for calcium gluconate, 2.20%–3.37% for adrenergic/dopaminergic agents, 3.13%–3.76% for glucocorticoids, and 1.05%–1.32% for antihistamines. These results are closer to the ADE rates reported through active pharmaceutical care (Hu et al., 2022) and suggest reduced actual occurrence versus spontaneously reported iodixanol-induced allergic reaction proportions. Therefore, analyzing adverse reaction signals in drugs after exposure using PSSA-like medical big data technology can better reveal real-world adverse reaction rates.

## 5 Limitations and strengths

Some assumptions were made to facilitate the methodology, including patients using iodixanol for the first time and only once. However, approximately 8% actually used it more than twice but were excluded, implying the theoretical ability to have reactions. Furthermore, adrenergic/dopaminergic agents and glucocorticoids have many clinical indications. Applying PSSA alone to ascertain allergic reactions, particularly acute ones, and inferring post-intervention adverse event proportions like drugs and surgery may be inappropriate. In this study, it was difficult to distinguish the reasons for drug use when processing big data. Since the prescription date accuracy in the Medicare database is only at the day level, the first prescription dates of the labeled drugs and indicator drugs could not be definitively determined as the same date. In classical PSSA, patients prescribed both drugs on the same day are usually excluded. For inpatient exposed drug adverse event monitoring, later reaction signal tracking like delayed hypersensitivity with ≤7-day intervals is more suitable. Adding a matched blank cohort without iodixanol exposure would improve this, comparing those receiving anti-allergics without iodixanol. This is the next step for further study and refinement.

## 6 Conclusion

To the best of our knowledge, this first multi-center Chinese inpatient database study detected iodixanol allergy signals, elucidating the applicability of different anti-allergic drug classes for signal detection and associated parameter settings. Meanwhile, inpatient iodixanol allergic reactions likely occur at substantially higher frequencies than those reported in collected spontaneous reports. We calculated the real-world iodixanol risk exposure and obtained results more closely aligned with actual pharmaceutical care findings. We also found that due to inpatient recording and reaction traits, PSSA is better suited for monitoring delayed hypersensitivity signals at intervals ≤7 days.

## Data Availability

The raw data supporting the conclusion of this article will be made available by the authors, without undue reservation.
